# Long-term outcomes of a Caucasian cohort presenting with acute coronary syndrome and/or out-of-hospital cardiac arrest caused by coronary spasm

**DOI:** 10.1007/s12471-017-1065-1

**Published:** 2017-12-13

**Authors:** W. Vlastra, M. Piek, M. A. van Lavieren, M. E. J. C. Hassell, B. E. Claessen, G. W. Wijntjens, T. P. van de Hoef, K. D Sjauw, M. A. Beijk, R. Delewi, J. J. Piek

**Affiliations:** AMC Heart Center, Academic Medical Center, University of Amsterdam, Amsterdam, The Netherlands

**Keywords:** Vasospastic angina, Coronary vasospasm, Acute coronary syndrome

## Abstract

**Background:**

Coronary artery spasm may be the underlying mechanism in up to 10% of cases of acute coronary syndrome (ACS) and sudden cardiac death. Asian individuals exhibit a 3-times greater incidence of spasm than Caucasians; this is likely due to different types of mechanisms. Consequently, solid data is limited about the long-term prognosis in Caucasian patients presenting with ACS and/or out-of-hospital cardiac arrest (OHCA) caused by coronary spasm.

**Methods:**

Between 2002 and 2015, thirty Caucasian patients with coronary artery spasm presenting with ACS (*N* = 29) and/or OHCA (*N* = 11) were enrolled in this prospective registry. Follow-up, consisting of regular outpatient visits, was conducted with a mean follow-up period of 7.5 ± 3.3 years. Outcomes included presence of stable angina pectoris, recurrence of ACS, occurrence of implantable cardioverter defibrillator (ICD) shocks and death.

**Results:**

The majority of patients (60%) remained asymptomatic during the entire follow-up period. At the end of the follow-up period only 3 patients still experienced stable angina (10%). Only 2 patients (7%) had a recurrent cardiac event, in which the ICD provided appropriate shock therapy. Half of the patients treated with stenting (*N* = 6), required re-interventions.

**Conclusion:**

Coronary spasm with ACS and/or OHCA in a Caucasian patient cohort has a relatively benign prognosis in the majority of patients in long-term follow-up, if treated appropriately with medical therapy. Both the role of ICD in OHCA secondary to coronary spasm, and the efficacy of stenting to treat vasospastic angina, warrant further study in large-sized prospective clinical trials.

## Background

Coronary artery spasm may be the underlying mechanism in up to 10% of cases of ischaemic heart disease, such as unstable angina pectoris, acute myocardial infarction and sudden cardiac death [1]. Coronary spasm is defined as an abnormal contraction of a localised segment of an epicardial artery (focal spasm), a dispersed contraction (diffuse spasm), contraction of two or more segments of the same artery (multifocal spasm) or multiple coronary branches (multivessel spasm) resulting in a dramatic reduction of coronary blood flow, generating myocardial ischaemia [2]. Vasospasm may occur at sites of focal stenosis, but is often documented in patients with apparently normal vessels at angiography [3]. The typical syndrome caused by coronary spasm is vasospastic angina, which most often occurs at rest and at night (Prinzmetal’s variant angina) [4], but can also be triggered by effort or stress conditions [5].

Vasospastic angina in Caucasian populations is relatively uncommon, it is the underlying cause of a mere 1.5% of all hospital admissions for (suspected) coronary artery disease [6]. In contrast, Asian individuals exhibit a 3-times greater incidence of spasm than Caucasian patients [7, 8]. The pathogenic substrate of coronary spasms is multifactorial [2]. Therefore, it is likely there is a difference in distribution of these pathogenic substrates between people with an Asian or Caucasian genetic background. Consequently, we can reasonably assume that there is an interracial difference in prognosis. Due to the low prevalence of vasospastic angina in Caucasian populations, limited data are available on the long-term follow-up of Caucasian patients with vasospastic angina, in particular in patients presenting with serious complications of vasospastic angina, such as unstable angina pectoris, non-ST-segment-elevation myocardial infarction (NSTEMI), ST-segment-elevation myocardial infarction (STEMI) and/or out-of-hospital cardiac arrest (OHCA). Therefore, the objective of this study was to report and evaluate the long-term outcomes of Caucasian patients diagnosed with acute coronary syndrome (ACS) and/or out-of-hospital cardiac arrest (OHCA) secondary to vasospastic angina.

## Methods

### Study design and patient population

The study was designed as a prospective single-centre registry. We included a total of 30 consecutive patients meeting the inclusion criteria presenting at the Academic Medical Center (AMC), University of Amsterdam, between 2002 and 2015. Patients presenting with an acute coronary syndrome and/or an out of hospital cardiac arrest, caused by vasospastic angina were enrolled. All included patients were suspected of vasospastic angina because of their clinical presentation, in combination with an absence of obstructive coronary artery disease at angiography. Per protocol, all patients suspected of coronary spasm underwent acetylcholine testing. We did not test patients with evident coronary spasm prior to the acetylcholine testing were not tested. Evident coronary spasm was considered present in case of documented transient ischaemic electrocardiographic signs in combination with the absence of obstructive coronary artery disease, or spontaneous spasm during diagnostic angiogram or provoked by wire manipulation at the onset of acetylcholine testing. Patients with non-Caucasian ancestry were excluded from the current study, due to suspected different pathogenic substrates.

### Cardiac catheterisation

Coronary angiography was performed through the femoral or radial approach according to operators’ preference using a 6 French sheath. We started the procedure with the administration of 5,000 i. e. units of heparin.

### Acetylcholine testing

**Fig. 1:**
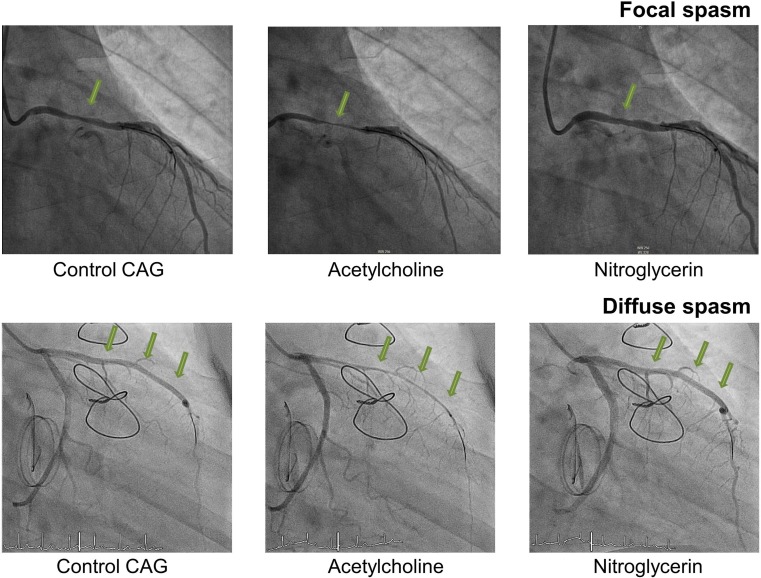
Images of coronary spasms, *CAG* coronary angiogram

Coronary spasm can be provoked by acetylcholine, which under normal circumstances dilates blood vessels [9]. However, in coronary arteries with endothelial dysfunction, acetylcholine induces a vasoconstrictive response [10]. In case of an elective acetylcholine test, vasodilators were discontinued 24 h before the cardiac catheterisation. Acetylcholine chloride was infused in continuous incremental doses (Table 1), into either the right coronary artery or the left coronary artery depending on the clinical presentation, until a spasm was provoked. In addition, if the first infusions did not provoke spasm, the maximum dose was provided. According to our institutional protocol, a Doppler guide wire (either FloWire of ComboWire, both Volcano, Rancho Cardova, Ca.) was introduced in the selected coronary artery to document flow alterations after acetylcholine testing and the early detection of cessation of blood flow due to coronary vasospasm. A positive response to acetylcholine testing was defined as transient occlusion (>90% narrowing) of a coronary artery with concomitant signs and symptoms of myocardial ischaemia (ST changes/angina). Fig. 1 displays examples of focal and diffuse coronary spasm in the current patient cohort.

**Table 1:** Protocol invasive coronary reactivity testing using acetylcholine

	Dose ACh (mg/ml)	Duration (min)	Infusion speed (ml/hour)
Dose 1 “Low”	0.000211	3:00 min	82 ml/hour
Dose 2 “Medium”	0.002105		
Dose 3 “High”	0.021053		
Dose 4 “Provocation”	0.210526		

Both calcium channel blockers and nitrates are discontinued 24 h prior to coronary reactivity testing. The different doses of acetylcholine are administered intracoronary

*ACh* acetylcholine

### Definitions

The Coronary Vasomotion Disorders International Study group (COVADIS) published the three core elements required to diagnose vasospastic angina [11], namely, transient ischaemic electrocardiographic changes, nitrate-responsive angina, and angiographic evidence of coronary artery spasm. Vasospastic angina can also be diagnosed without angiographic confirmation when an episode of spontaneous angina, associated with transient ST-segment elevation, that promptly reacts to short-acting nitrates is observed.

### Clinical follow-up and outcomes

We collected baseline demographic variables, procedural and angiographic characteristics in a dedicated electronic database. Follow-up was conducted actively in all patients and consisted of regular outpatient visits to the cardiologist. Main outcomes collected during these visits included the presence of stable angina pectoris, recurrence of ACS, occurrence of implantable cardioverter defibrillator (ICD) shocks and re-interventions. In case of admissions in other hospitals, source documents were retrieved.

### Statistical analysis

We reported patient characteristics as percentages, counts or mean ± standard deviation (SD), where appropriate. Analyses were performed using IBM SPSS version 23.0 software (SPSS Inc, Chicago, IL).

## Results

### Patient characteristics

The patient characteristics of the study population (aged 56 ± 9 years, 50% women) are displayed in Table 2. In total 8 patients presented with unstable angina pectoris (27%), 3 with NSTEMI (10%), 18 with STEMI (60%) and/or OHCA (*N* = 11, 24%).

**Table 2:** Baseline patient characteristics and procedural and angiographic data

	*N* = 30
**Demographics**	
Age	56 (±9)
Male	15 (50%)
Caucasian	30 (100%)
**Risk factors/Medical history**	
Hypertension	11 (37%)
Dyslipidaemia	9 (30%)
Diabetes Mellitus	0 (–)
Smoking	7 (24%)
Ex-smoker	6 (20%)
– Family history of ischaemic heart disease	8 (27%)
– Previous MI	8 (27%)
**Presentation of coronary spasm**	
Unstable angina	8 (27%)
STEMI	18 (60%)
NSTEMI	3 (10%)
OHCA	11 (37%)
***Clinical situation during presentation***	
In rest	25 (83%)
Physical effort or emotional distress	5 (17%)
**Angiographic characteristics**	
IVUS/OCT use	16 (53%)
***Coronary stenosis***	
Without stenosis	15 (50%)
Non-significant^a^	15 (50%)
Significant	0 (–)
***Positive acetylcholine provocation test***	17 (57%)
Single vessel spasm (*N* = 11)	
– LAD	7 (64%)
– RCA	3 (27%)
– CX	1 (3%)
Multivessel spasm (*N* = 6)	
***Spontaneous spasm-positive artery***	7 (23%)
Single vessel spasm (*N* = 5)	
– LAD	3 (60%)
– RCA	2 (40%)
– CX	0 (–)
Multivessel spasm (*N* = 2)	
***Spasm not objectified by angiography*** ^b^	6 (20%)
***Type of spasm (N =*** ***24)***	
Diffuse	13 (54%)
Focal	11 (46%)
Multifocal	0 (–)
**Treatment**	
ICD	8 (27%)
Stenting (N = 6)	6 (20%)
– LAD	4 (67%)
– CX	1 (17%)
– RCA	1 (17%)
***Medical treatment at discharge***	
Calcium channel blocker	26 (87%)
Long-acting nitrate	13 (43%)
Antiplatelet	28 (93%)
Statin	22 (73%)
ACEI/ARB	17 (57%)
Beta-blocker	1 (3%)

*ACEI* angiotensin-converting-enzyme inhibitor, *ARB* angiotensin receptor blockers,* Cx* circumflex artery*, ICD* implantable cardioverter defibrillator,* IVUS* intravascular ultrasound, *LAD* left anterior descending coronary artery, *MI* myocardial infarction, *NSTEMI* non-ST-elevation myocardial infarction, *RCA* right coronary artery, *OHCA* out-of-hospital cardiac arrest*, OCT* optimal coherence therapy,* STEMI* ST-elevation myocardial infraction

^a^Non-significant stenosis defined as 25–50% diameter stenosis

^b^In 6 patients, coronary spasm was considered because of dynamic electrocardiographic changes and/or positive cardiac enzyme release in the presence of angiographically normal coronary arteries. Acetylcholine provocation testing was not performed in these patients

### Documentation of vasospastic angina

In 17 patients (57%) vasospastic angina was documented by a positive acetylcholine test. In 7 patients (23%) vasospastic angina was documented either by a spontaneous episode of coronary spasm at angiography or induced after the introduction of a guide wire prior to acetylcholine testing. In the remaining 6 patients (20%) vasospastic angina was considered present because of dynamic electrocardiographic changes and/or positive cardiac enzyme release in the presence of angiographically normal coronary arteries. In these 13 patients, acetylcholine testing was considered redundant.

### Angiographic characteristics

**Fig. 2:**
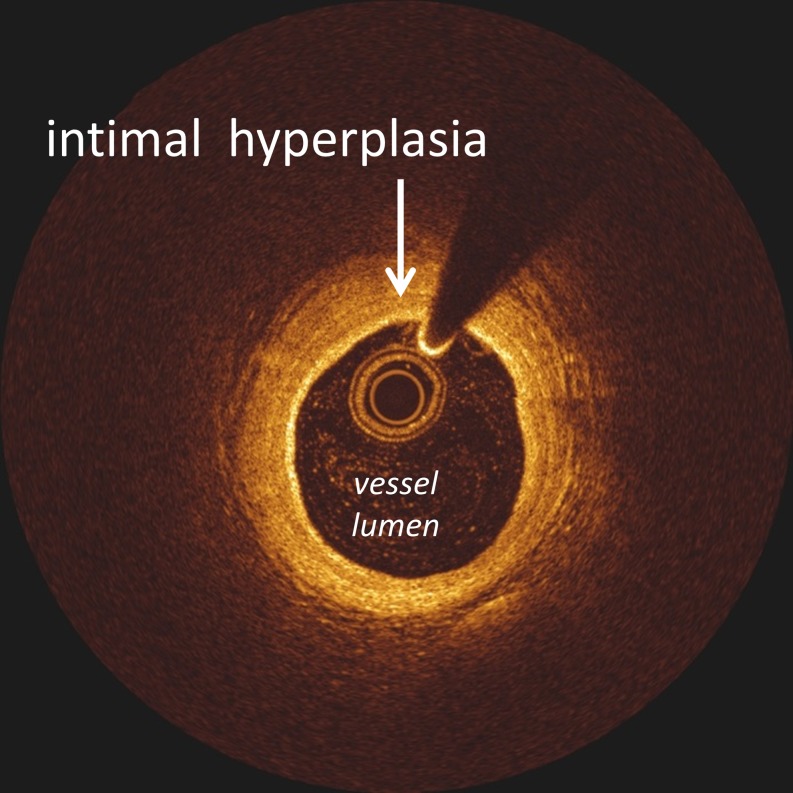
Image of intimal hyperplasia

Patients with an angiographically documented coronary spasm, showed a single vessel spasm in 16 patients versus multivessel spasm in 8 patients. Both in acetylcholine provocation testing of the left coronary artery and in case of a spontaneous spasm, the left anterior descending artery was predominantly affected (Table 2). The distribution of patients presenting with either diffuse spasm or focal spasm was comparable (54% versus 46%). Furthermore, in the total study cohort we found non-significant atherosclerotic coronary stenosis in 15 patients (50%). Additional intravascular imaging using intravascular ultrasound (IVUS) or optimal coherence therapy (OCT) was performed in 16 (53%) of the patients and showed intima hyperplasia or minimal atherosclerotic disease (Fig. 2).

### Treatment

The treating physicians prescribed calcium channel blockers in 26 (87%), long-acting nitrates in 13 (43%), anti-platelet therapy in 28 (93%), statins in 22 (73%) and ACE-inhibitors in 17 (57%) patients to treat the vasospastic angina. A beta-blocker was prescribed in one patient (3%) with persisting ventricular arrhythmias, despite a general contraindication for beta-blockers in patients with coronary spasm. Out of 11 patients who presented with OHCA, 8 (73%) received an ICD. In one patient, the treating physician deemed ICD implantation to be unnecessary since the cardiac arrest was evidently provoked by surgery of a neuro-endocrine tumour. In the remaining two patients initially presenting with anteroseptal myocardial infarction and primary ventricular fibrillation, the responsible clinicians considered ICD implantation to be non-beneficial. Additionally, 6 patients (13%) were treated with stent implantation. All six patients had documented vasospasm as well as non-significant stenosis of the same segment, in combination with ongoing angina, not responsive to medication.

### Long-term clinical outcomes

Patients were followed for a mean of 7.5 ± 3.3 year (Table 3). During follow-up, 12 patients (40%) suffered from stable angina pectoris. In only 3 patients (10%) the angina persisted until the end of the follow-up period. More than half of the patients (53%) did not experience angina, encountered no cardiac events and received no appropriate ICD shock during the entire follow-up period. In 2 patients who presented with an OHCA, ventricular fibrillation occurred during follow-up, in both cases the ICD provided appropriate shocks. Appropriate antiarrhythmic medication was not adequately installed in one patient, while the other patient was suspected of ongoing cocaine abuse. Additionally, one patient who was asymptomatic during the entire follow-up period received multiple inappropriate ICD shocks. Of the 6 patients treated with stent implantation, 3 were asymptomatic after treatment. The remaining 3 patients required re-interventions due to new spasm in the segment adjacent to the implanted stent (*N* = 2) or in-stent-restenosis (*N* = 1). One patient died of non-cardiac disease within the follow-up period.

**Table 3:** Follow-up

	***N*** **= 30**
**Events**	
Asymptomatic^a^	16 (53%)
*Vasospastic angina*	
During follow-up period	12 (40%)
During the last year of the follow-up period	3 (10%)
*Death*	1 (3%)
Cardiac death	0 (–)
*ICD shock (N = 8)*	3 (38%)
Appropriate shock	2 (25%)
*Re-intervention (N = 6)* ^*b*^	3 (50%)

*ICD* implantable cardioverter defibrillator

^a^The number of patients that were asymptomatic during the complete follow-up period (no angina, no cardiac events and no appropriate ICD shocks)

^b^Due to new spasm in the segment adjacent to the implanted stent (*N* = 2) or re-stenosis of bare metal stent (*N* = 1)

## Discussion

This present study shows, for the first time, the long-term clinical follow-up of Caucasian patients with ACS and/or OHCA as the initial presentation of vasospastic angina. Our results indicate a relatively benign course of this disease in most patients, if the disease is treated appropriately with medical therapy.

### Description of the cohort

The patient cohort described in this study is in line with previous studies of patients with vasospastic angina, showing endothelial dysfunction as determined by acetylcholine testing, in the presence of minimal or mild atherosclerotic disease in the majority of patients who underwent IVUS or OCT. The long-term follow-up of this patient cohort showed that a large part of the cohort remained symptomatic during the initial one or two years following presentation, while the symptoms disappeared beyond this period without relapse of ACS or OHCA. Current guidelines do not support stent implantation in vasospastic arteries without severe obstructive coronary narrowing, in particular in patients with severe organic stenosis [10]. Stent implantation is only indicated for those patients who are still symptomatic despite optimal medical therapy, and in combination with focal stenosis. Although the number of patients studied is small, it is an interesting observation that those patients treated with stent implantation often required re-intervention due to vasospasm in the adjacent segments of the stent implantation. This suggests that we should use stent implantation with caution. Treating physicians implanted ICDs in 8 patients with OHCA. Only two patients documented appropriate ICD shocks, shortly after the initial event. These events were presumably the consequence of cocaine abuse and insufficient antiarrhythmic control. Interestingly, none of the patients required additional ICD shocks after consistent administration of adequate therapy. Nevertheless, the sample size was too small to draw any conclusions regarding the benefit of ICD implantation.

### Comparison with the literature

The majority of published studies focus on the underlying mechanism of vasospastic angina in relation to the clinical presentation. The largest database on vasospastic angina in Caucasian patients comes from the WISE study group. They emphasise the pivotal role of endothelial dysfunction as the pathological substrate in coronary artery spasm, causing angina in the absence of epicardial disease [12]. Previous studies also evaluated the occurrence of cardiac events during long-term follow-up in patients diagnosed with vasospastic angina [13–16]. These studies concluded that cardiac events occurred more often in patients with endothelial dysfunction compared with patients without endothelial dysfunction. But the studies did not provide data on the use of calcium channel blockers [16], or treatment with calcium channel blockers was not installed in the majority of patients [13]. Additionally, the studies showed that gender and age differences play an important role in the long-term outcomes of vasospastic angina [14]. Moreover, diffuse spasm was associated with a better prognosis than focal spasm.

Despite these follow-up studies, limited information is available on the long-term follow-up of those patients who presented with serious complications of vasospastic angina, such as ACS. Few studies have addressed the prognosis of patients with vasospastic angina presenting with a cardiac arrest, these studies have different outcomes, small sample sizes and/or short follow-up periods [17–20]. In a recent study by Ahn et al. [21], long-term mortality and ventricular tachyarrhythmic events in patients with aborted sudden cardiac death (ASCD) was investigated in a Korean population. They concluded that the prognosis of patients with vasospastic angina with ASCD was worse than other patients with variant angina, despite optimal medical treatment. Nevertheless, their study addressed a different population than the current study. First, their study solely included patients presenting with cardiac arrest, whereas the current study also enrolled patients with an acute coronary syndrome, without a subsequent cardiac arrest. Second, in the study by Ahn et al. only 13% of the patients received an ICD, even though implantation is recommended in patients with an episode of resuscitated ventricular tachycardia/ventricular fibrillation and a cause that is not completely reversible. Therefore, this might have led to a higher mortality. Particularly, since they described a nonsignificant trend of a lower rate of cardiac death in patients who did receive ICD implantation. Third, the study by Ahn et al. obtained 8 years follow-up from only 20% of the enrolled population with ASCD. In addition, another recent retrospective Korean cohort study by Cho et al. [22] described the follow-up in a large cohort of patients with vasospastic angina, as diagnosed with ergonovine provocation testing, presenting with ACS versus patients presenting without ACS. The study concluded that patients initially presenting with ACS had an increased risk of recurrent myocardial infarction and rehospitalisation because of recurrent angina. However, both studies address the Korean population, in which vasospastic angina has a higher incidence than in Caucasian populations. A different pathophysiology could not be ruled out [7]. This might explain the higher relapse rate of cardiac events.

### Comparison of the cohort with patients with obstructive coronary artery disease

The low rates of recurrent cardiac events (1 in 15 patients) in this study cohort stands in stark contrast to the high rates of recurrence in patients with ACS and/or OHCA caused by structural coronary artery disease. In the current study population, most patients presented with STEMI and were treated conservatively. Nevertheless, at the end of the follow-up period, mortality and recurrence of cardiac events were very low. In contrast, in the 5‑year follow-up of the ICTUS trial [23], 1 out of 5 patients with non-ST-segment-elevation ACS treated with a selective invasive strategy had experienced a myocardial infarction or died. This underlines the difference between obstructive coronary artery disease versus non-obstructive coronary artery disease.

### Coronary spasm provocation testing

In the setting of endothelial dysfunction, provocation testing causes contraction of smooth muscle cells. Provocation testing to diagnose coronary spasm is not only important to reassure the patient that a cause for the ACS is found, it also allows the physician to provide adequate medical therapy (starting calcium channel blockers and statins, possibly nitrates and avoiding beta-blockers) and strongly stimulate cessation of smoking. Almost one-third of the patients presenting with ACS has unobstructed coronary arteries during coronary angiography (CAG) [1]. Almost half (48%) of the patients with ACS and unobstructed coronary arteries, show coronary spasm during intracoronary acetylcholine testing. Ergonovine testing is also frequently used to provoke coronary spasm during CAG. However, ergonovine is suspected to be less sensitive, resulting in false-negative outcomes of the test [24, 25]. Additionally, acetylcholine testing is relatively safe with complications (non-fatal arrhythmias) occurring in 1% [25, 26]. Alternatively, coronary spasm can be provoked with the cold pressor test, atrial pacing and exercise and hyperventilation. Nevertheless, the sensitivity and specificity of these tests are lower than ergonovine and acetylcholine testing and therefore of limited diagnostic value [27, 28]. In short, we recommend acetylcholine testing in all patients presenting with ACS who show unobstructed coronary arteries during CAG.

### Study limitations

The first study limitation is that acetylcholine testing was not performed in all studied patients. Nevertheless, coronary spasm was considered present because of dynamic electrocardiographic changes and/or positive cardiac enzyme release in the presence of angiographically normal coronary arteries. Also, the number of patients studied was relatively small, although these patients were followed for a lengthy period (a mean follow-up period of almost 8 years). Subsequently, the current findings need to be verified in a large prospective clinical trial.

## Conclusion

The present study showed a relatively benign prognosis during long-term follow-up in a majority of the Caucasian patients with coronary artery spasm, presenting with ACS and/or OHCA. Both the role of ICD in OHCA secondary to coronary spasm and the efficacy of stenting to treat vasospastic angina warrant further study in large-sized prospective clinical trials.
